# Thirty years of CMV seroprevalence—a longitudinal analysis in a German university hospital

**DOI:** 10.1007/s10096-020-03814-x

**Published:** 2020-01-27

**Authors:** Sebastian Hoehl, Annemarie Berger, Sandra Ciesek, Holger F. Rabenau

**Affiliations:** Institute for Medical Virology, University Hospital, Goethe University Frankfurt am Main, Paul-Ehrlich-Straße 40, 60496 Frankfurt am Main, Germany

**Keywords:** Cytomegalovirus (CMV), Seroprevalence, Anti-CMV IgG, Human immunodeficiency virus (HIV), Congenital CMV infection, CMV epidemiology

## Abstract

Human cytomegalovirus (CMV) is a significant cause of morbidity and mortality in patient groups at risk. We have previously shown that the anti-CMV IgG seroprevalence in an urban region of Germany has changed over the last decades. Overall, a decline from 63.7 to 57.25% had been observed between 1988–1997 and 1998–2008 (*p* < 0,001). Here, we continuously follow the trends to the most recent decade 2009 to 2018. In a retrospective analysis, we determined the seroprevalence of CMV IgG antibodies in our patient cohort, stratified by gender and selected groups at risk (e.g., patients with HIV infection; women of childbearing age). The overall prevalence of anti-CMV IgG non-significantly declined further from 57.25% in 1998–2008 to 56.48% in 2009–2018 (*p* = 0.881). Looking at gender differences, overall CMV seroprevalence in males declined to 52.82% (from 55.54% in 1998–2008; *p* = 0.0254), while it non-significantly increased in females to 59.80%. The high seroprevalence in patients with a known HIV infection further increased from 87.46% in 1998–2008 to 92.93% in the current period (*p* = 0.9999). In women of childbearing age, no significant changes over the last three decades could be observed. The CMV seroprevalence in oncological patients was determined to be 60.64%. Overall, the former significant decline of CMV seroprevalence between the decades 1988–1997 and 1998–2008 in this urban region of Germany slowed down to a non-significant decrease of 0.77% (1998–2008 vs. 2009–2018). This might be an indicator that CMV seroprevalence has reached a plateau.

## Introduction

Primary CMV infection in immunocompetent hosts may be asymptomatic, or may cause mostly mild, self-limiting disease with fever, fatigue, headaches, and myalgia [1, 2]. After primary infection, the virus remains latent. Infants and toddlers are an import source of infection, as they can shed the virus by urine or saliva for months or even years after infection [3, 4].

Primary infection, reactivation, or reinfection shortly before or during pregnancy can result in congenital CMV infection, which is estimated to lead to permanent disability in 1 to 2 cases per 1000 pregnant women in Germany [5], making it the most common infectious cause of birth defects. Permanent sequelae include sensorineural hearing loss (SNHL), microcephaly, seizures, neurologic deficits, and retinitis. Counseling about hygiene can lower the risk of anti-CMV IgG negative women to get infected during pregnancy [5, 6]. The rate of SNHL appears to be similar for children born after primary and non-primary maternal CMV infection, but seems to be more severe in the primary maternal infection group [7].

In immunocompromised hosts, CMV poses a major burden of disease. People living with HIV/AIDS usually have a high rate of CMV seropositivity. Therefore, the risk of primary CMV infection is low, but reactivation or reinfection as an opportunistic infection most commonly manifests as retinitis, colitis or esophagitis, hepatitis, encephalitis, myeloradiculopathy, or pneumonia. Otherwise immunocompromised hosts, such as oncological patients and solid organ or stem cell transplant recipients, are additional groups affected by increased risk of morbidity and mortality associated with CMV.

Insight into the dynamics of CMV epidemiology is therefore highly relevant for diverse, vulnerable groups of patients.

Different areas of the world can be divided in low (50 to 70%) and high seroprevalence settings (> 70%) [8]. It is usually higher in low income countries [9–14]. A limited number of studies have focused on the CMV seroprevalence in Germany. In children and adolescents in Germany, a study of the CMV IgG seroprevalence (KiGGS study, 2003–2006) found a range between 21.4% in boys at 1–2 years of age and 33.5% in girls at 14–17 years of age. CMV seroprevalence increased with age in both genders. Risk factors for higher seroprevalence included migration background, place of birth other than Germany, having attended daycare, and having younger siblings [15]. In a study in the adult population of Germany, sera collected throughout Germany in 1998, published 2018, the overall CMV seroprevalence was 56.7% and also increased with age. It was higher in females (62.3%) than in males (51.0%). Total seroprevalence in women of childbearing age (18 to 45 years of age) was 51.7%. The study also identified risk factors associated with seropositivity, with country of birth and age being the strongest independent factors [11].

The comparative changes of CMV seroprevalence among the patients of the urban University Hospital of Frankfurt, Germany, over the period of 1988 to 1997 and 1998 to 2008 have previously been assessed. An overall decline in CMV seroprevalence among its patients without a known HIV infection from the first to the second decade, decreasing from 63.70% to 57.25%, was observed. This decrease in CMV seroprevalence has also been observed in other studies in Germany [13, 16] and, in some groups, in Spain [14], but not in the USA [12]. The decrease has also been observed in bone marrow donors in Germany [16]. In the former study conducted in Frankfurt, females had a slightly higher CMV seroprevalence than men (63.83% compared with 65.54% in 1988–1997 and 58.73% compared with 55.54% in 1998–2008). Patients with a known HIV infection had a significantly higher CMV seroprevalence (in females, 83.17% in 1988–1997 and 87.80% in 1998–2008, in males, 88.76% in 1988–1997 and 87.32% in 1998–2008) [13].

In the current study, we set out to determine whether the decline continued to the most recent decade, and especially observed the changes over in time in two selected groups of vulnerable patients, the developing fetus, examined in proxy by the status of their pregnant mothers, as well as people living with HIV/AIDS.

As another important group affected by CMV disease, we also looked at oncological patients. We determined the accumulated CMV seroprevalence of those patients whose samples were sent in from oncology wards, stratified by pediatric and adult patients.

## Material and methods

We conducted a retrospective analysis of the data routinely acquired during patient care at the University Hospital of Frankfurt. All results of anti-CMV IgG antibody tests from our Institute of Medical Virology in the time frame January 1, 2009, and December 31, 2018, were analyzed. The University Hospital of Frankfurt is the largest hospital and a maximum care provider in Germany’s fifth largest city. It treats about 49,000 in-patients and 229,000 out-patients a year.

All anti-CMV IgG tests (Enzygnost anti-CMV IgG, Dade Behring, Marburg, Germany) were performed on the Behring ELISA Processor BEP 2000, the results were recorded semi-quantitatively as arbitrary units per milliliter (AU/mL). The test results of the decade 2009–2018 were compared with the two decades of the former study (1988–1997; 1998–2008) [13] in which the same diagnostic test had been used.

### Patient collective

Due to the possibility of maternal antibody detection, infants below the age of 1 year were excluded. Of each patient, only the first CMV IgG test in the time period was considered, and seropositivity defined as a positive result in the anti-CMV antibody IgG assay. Patients with an unspecific or borderline reactivity on the CMV IgG test were excluded. Group, age- and gender-dependent analysis was performed. The mathematical median of the positive test results was calculated.

Patients with a confirmed HIV infection were analyzed separately to avoid bias by a higher-than-average CMV seroprevalence within this group and overrepresentation in our patient cohort. Positive HIV status was defined as a positive result in a 4th generation antibody/antigen-HIV screening test followed by a positive confirmation test by HIV immunoblot and/or HIV-PCR in the studied time period.

The data of all three decades was revisited to determine the CMV IgG seroprevalence of women of childbearing age. We chose samples of women of childbearing age sent in by our Department of Obstetrics and Gynecology, as testing of those samples was presumed to be indicated by pregnancy or birth. We compared the CMV seroprevalence in this group between the three decades, and formed four age groups for comparative analysis. Patients younger than 16 or older than 45 years of age were excluded from age-dependent analysis, since there were a relatively low number of those samples, and therefore, CMV seroprevalence could not be determined with confidence. In addition, we determined the accumulated CMV seroprevalence of patients whose samples were sent in from an oncological department or ward. We stratified by pediatric and adult patients, defined by patient age. All patients younger than 18 years of age were considered to be pediatric.

Statistical testing was performed using BiAS® for Windows (version 11.10, epsilon-Verlag, Hochheim, Germany, 2019), including the calculation of the 95% confidence intervals. *P* values were calculated using the two-tailed, not Yates rectified chi-squared test. A *p* value of ≤ 0.05 was defined as statistically significant.

Figures were created using GraphPad Prism 6.

## Results

Samples of a total of 31,401 patients above the age of 1 year had been tested for anti-CMV IgG antibodies in the years between 2009 and 2018. A total of 258 patients who had borderline or unspecific test results were excluded, leaving 31,143 patients with an unambiguous result.

### All patients after exclusion of those known to be HIV positive

The overall CMV IgG seroprevalence in the cohort in the decade 2009–2018, after exclusion of 1740 patients with known HIV infection, was 56.48% (*n* = 29,403). The median value in the HIV-negative population was 1400 AU/mL.

When comparing anti-CMV IgG seropositivity to the data of the former decades, a continuous decline is visible: from 63.70% (*n* = 29,374) in the decade 1988–1997 and 57.27% (*n* = 20,397) in the decade 1998–2008.

**Fig. 1:**
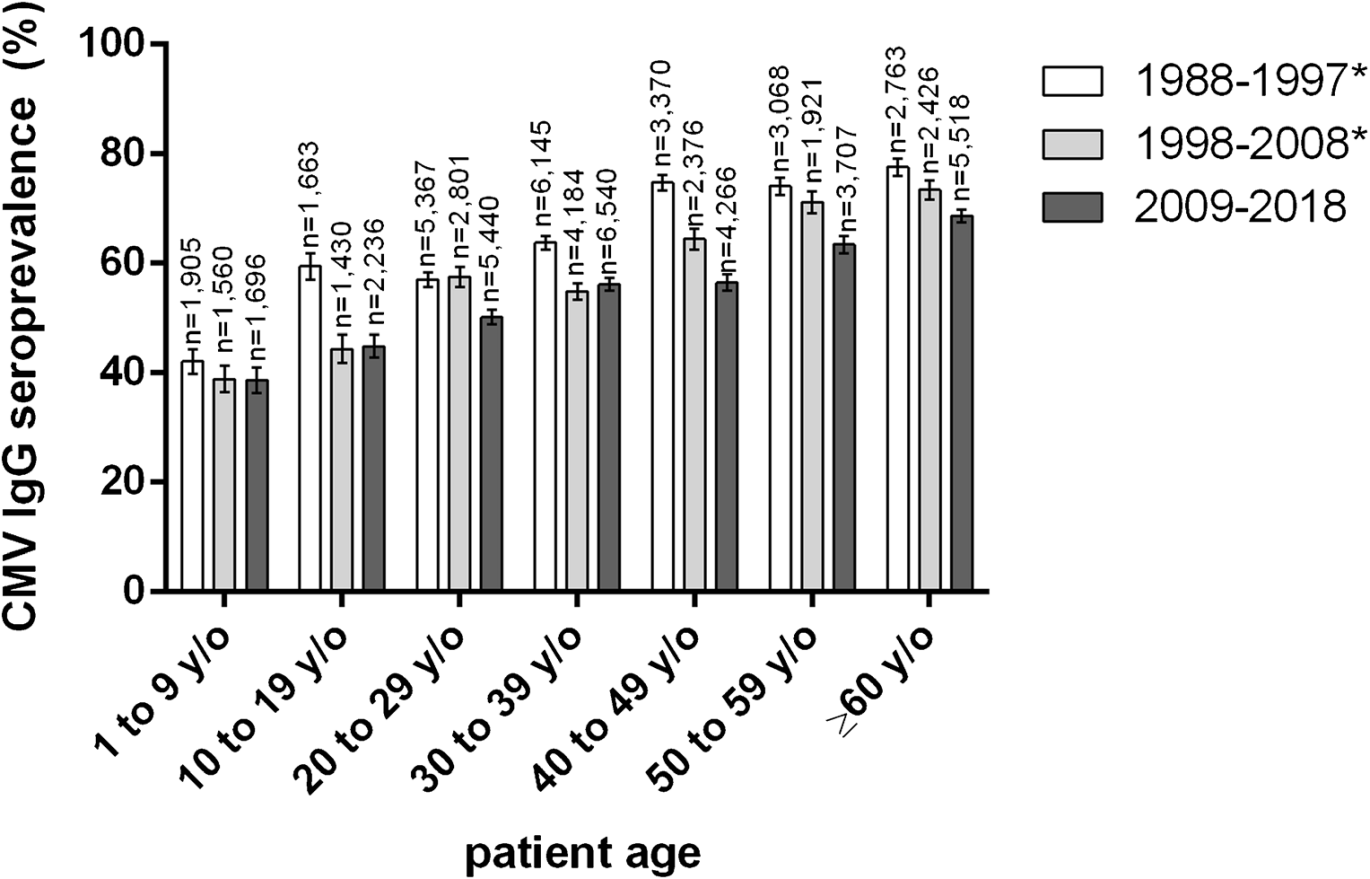
CMV IgG seroprevalence by age group and decade. Patients with a known HIV infection were excluded in all decades. *Data previously published by our group (Lübeck et al.) [13]; y/o, years old

While the slight overall decrease from the previous to the most recent decade (1998–2008 to 2009–2018) of 0.77% was not statistically significant (*p* = 0.0881), a significant decrease can confidently be assessed when looking at the age groups “20 to 29,” “40 to 49,” “50 to 59,” and “60 years of age and older,” respectively (*p* values < 0.0001) (Fig. 1). CMV seroprevalence throughout all age groups declined significantly when compared with the decade 1988–1997. A sustained decrease spanning all three decades can be observed in the group of 1 to 9 year olds, and in all age groups 40 years and older (Fig. 1).

### CMV seroprevalence by gender

**Fig. 2:**
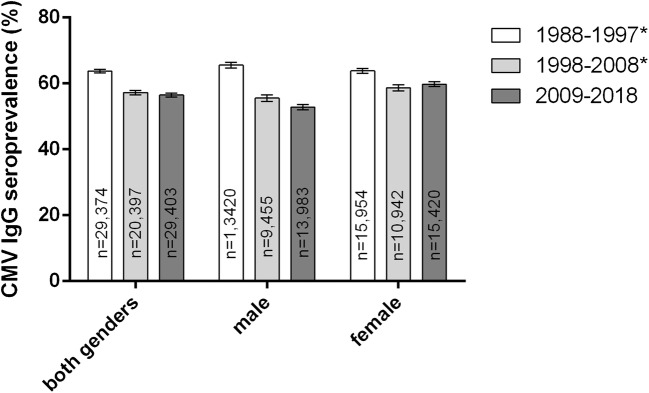
CMV IgG seroprevalence by gender and decade. *Data previously published by our group (Lübeck et al.) [13]

The overall CMV seroprevalence in the most recent decade 2009–2018 was significantly higher in females (59.80%, *n* = 15,420) than in males (50.82%, *n* = 13,983, *p* < 0.0001). During the studied period of 30 years, CMV seroprevalence decreased in both sexes. However, from the time period of 1998–2008 to 2009–2018, there was a non-significant increase in females (*p* = 0.0811) (Fig. 2), and a significant decrease of 2.72% in males (*p* = 0.0254).

**Fig. 3:**
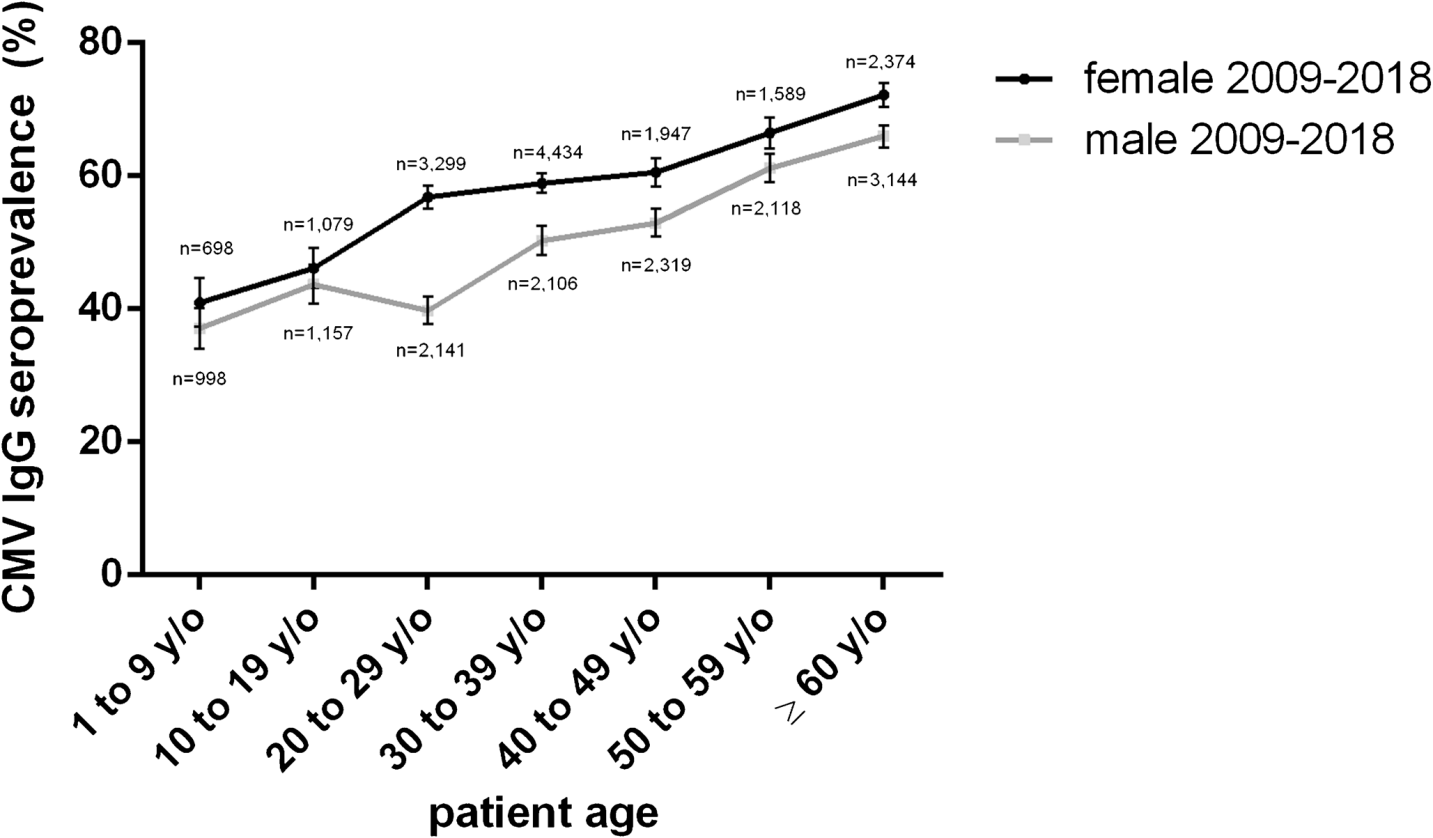
CMV IgG seroprevalence 2009–2018 by age group [13]

Looking at the most recent decade only, CMV seroprevalence is higher in females in all age groups, however, not statistically significant in the age groups 1 to 9 and 10 to 19 years of age (*p* = 0.1046 and *p* = 0.2503, respectively), while significant in all other age groups. In the group 20 to 29 years of age, there is a sharp increase in females of 10.69% and a decline in males, resulting in a large gap between the genders of 17.09% (39.75% in males, 56.84% in females). In males, CMV seroprevalence increases sharply between the age groups 20 to 29 and 30 to 39 years of age, by 10.58%. Finally, CMV seroprevalence in both sexes merge toward a linear increase, with seroprevalence in females about 7.5% higher than in men (Fig. 3).

### Women of childbearing age

**Fig. 4:**
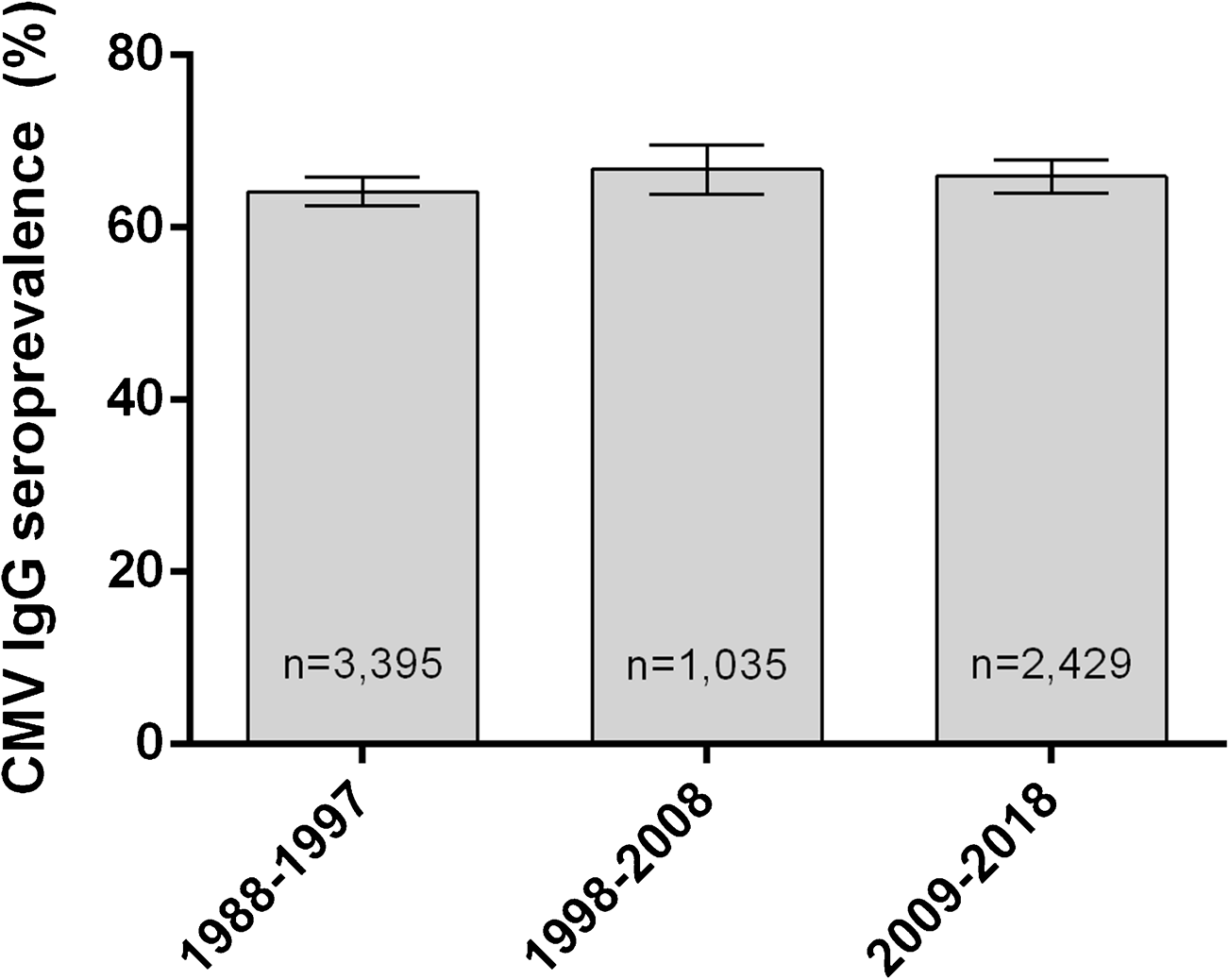
CMV IgG seroprevalence of women of childbearing age, by decade

There was no major change in the overall CMV seroprevalence of women of childbearing age presenting to the Department of Gynecology and Obstetrics during the last three decades. A slight increase from 64.18% (*n* = 3395) in the decade 1988–1997 to 65.95% (*n* = 2429) in 2009–2018 cannot be asserted with confidence (*p* = 0.1627) (Fig. 4).

**Fig. 5:**
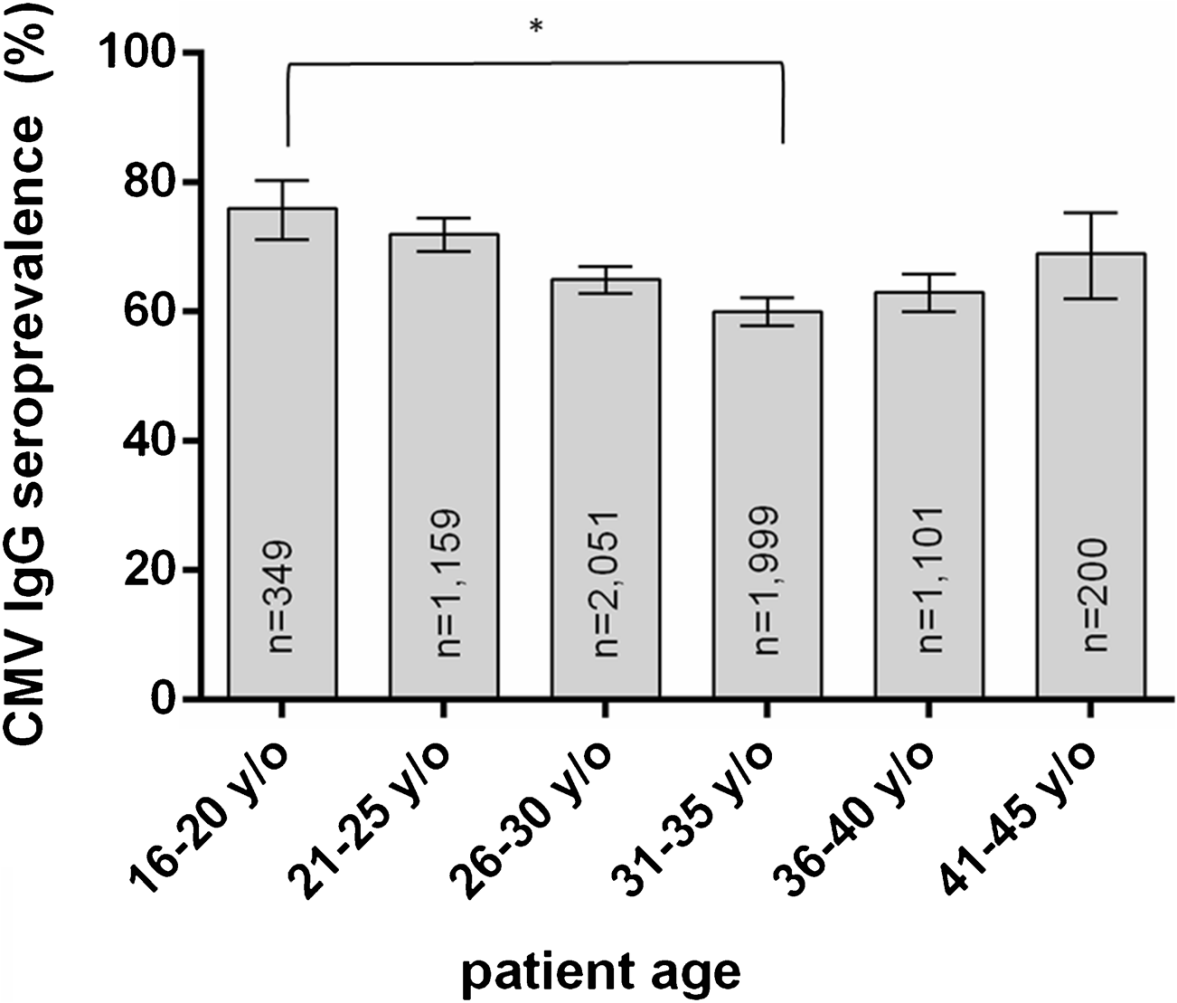
CMV IgG seroprevalence 1988–2019 by age group from women of childbearing age

A look at the distribution between different age groups spanning all three decades reveals the highest seroprevalence rate in the youngest age group (16 to 20 years of age), that is declining up to the age group 31 to 35 years of age, followed by a continuous increase with progressing age (Fig. 5). The decrease from 16 to 20 years of age (76.22%) to 31 to 35 years of age (60.13%) is statistically highly significant (*p* < 0.0001).

### CMV seroprevalence in HIV-positive patients

In our cohort, 1740 patients had a known HIV infection. Of those, 1617 had a positive anti-CMV IgG test result; the seroprevalence was 92.93%. The median of all positive samples was 2409 AU/mL. Compared with the data of the two previous decades, we could observe an increase in seropositivity of 5.47%, which is not statistically significant (*p* = 0.9999).

**Fig. 6:**
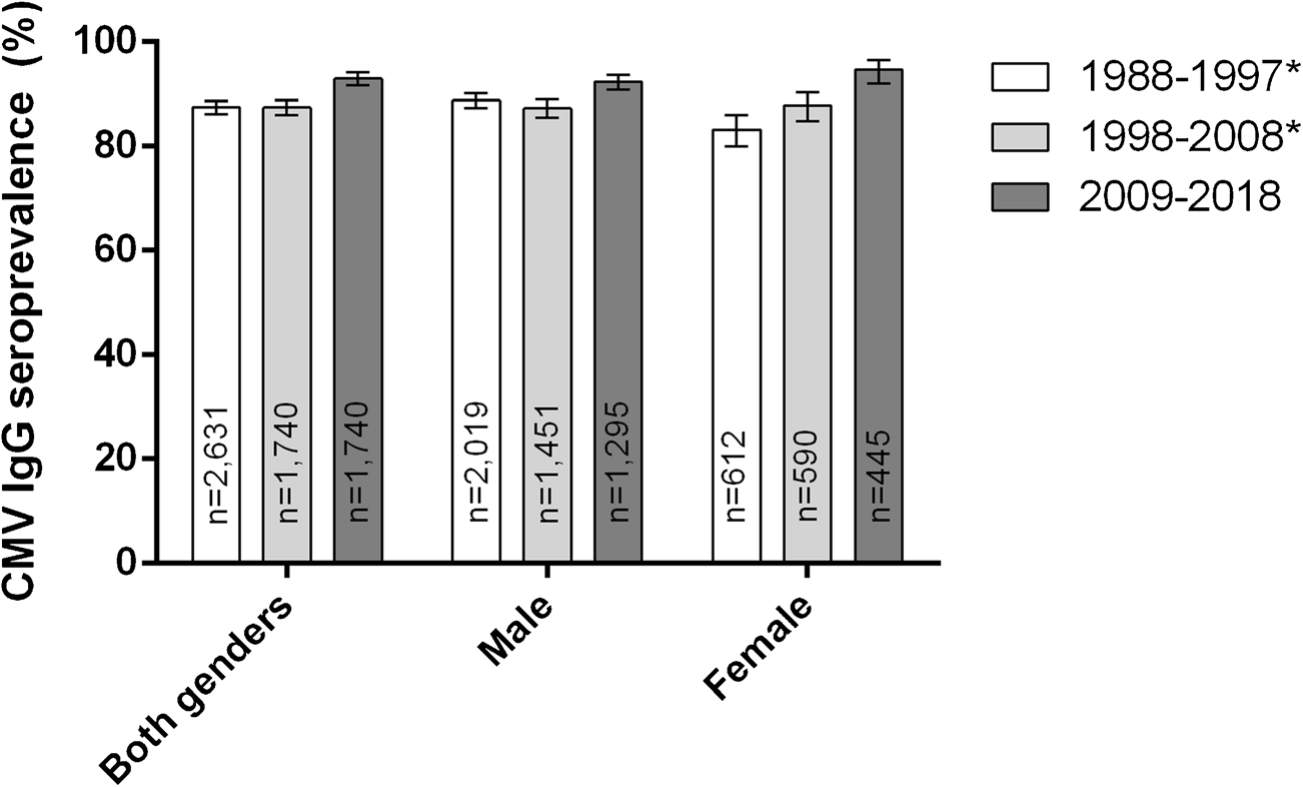
CMV IgG seroprevalence by gender and decade of patients with known HIV infection. *Data previously published by our group (Lübeck et al) [13]

When looking at the genders separately, the largest increase was in females with a known HIV infection, with an increase of 6.81% when compared with 1998–2008, and 11.44% when compared with 1988–1997. Between the former two decades, the increase had little confidence, since the 95% confidence intervals overlapped. When compared with 1988–1997, however, the increase is highly significant (*p* < 0.0001) (Fig. 6).

A significant difference in CMV seroprevalence between males and females with a known HIV infection had not been observed in the decades of 1988–1997 and 1998–2008. In the most recent decade, however, the CMV seroprevalence of females with a known HIV infection was higher than in males (94.61% and 92.36%, respectively), but with little confidence (*p* = 0.1099).

High CMV seroprevalence in patients with a known HIV infection could be observed in all age groups with little variation. In the analysis by sex and age in the group of patients with a known HIV infection, the sample size was too small to confidently identify differences.

### CMV seroprevalence in oncological patients

In patients whose samples were sent in from oncological departments, we determined a combined CMV seroprevalence of 60.64% (*n* = 2091). It was lower in the pediatric population, where it was 51.60% (*n* = 219), and higher in the adult population (61.70%, *n* = 1872).

## Discussion

In this study, we present an overview of the changes in CMV IgG seroprevalence of the patients of the urban University Hospital of Frankfurt over a time period of 30 years.

In the most recent decade (2009 to 2018), the overall CMV seroprevalence in patients without a known HIV infection was 56.48%. This number is within the wide range of the previously published data for population groups in Europe of 30.4 to 89.7%. [9, 11, 13–19]. The slight decrease of 0.77% from the previous decade (1998 to 2008) was not statistically significant (*p* = 0.0881). Therefore, the decline in overall CMV seroprevalence in Germany, that has also been observed in other studies [16], appears to be slowing down or even halting. Changes in lifestyle, such as well-documented tendency toward smaller households [20] in Germany, with fewer young children as possible sources of infection [21] during the last decades, may have been a driver behind the decreasing trend, which was continuously observed in the age group of 20 to 29 years of age and all groups above the age of 40 years. In our youngest patients, below the age of 20 years, and in the age group 30–39 years, however, the trend halted or even reversed. In the youngest patients, this might be associated with more children below the age of 3 years attending daycare, where the exposure to potentially infectious toddlers as a source of infection is increased, a known factor associated with CMV seropositivity in Germany [15]. Notably, from 2006 to 2017, the percentage of children below the age of 3 years attending day care in Germany increased from 13.6 to 33.1% [22]. Another factor offsetting the decreasing trend in CMV prevalence might be associated with increased migration from non-EU countries, especially to the urban centers of Germany. This could most markedly affect the age group with the largest number of patients: 30 to 39 year olds saw an increase of 1.28% in CMV seroprevalence (Fig. 3). Non-EU nationals currently make up 22.08% of all 25- to 44-year olds living in Frankfurt. Non-EU nationals living in Frankfurt largely stem from regions with high CMV seroprevalence, most markedly Turkey [23], where CMV seroprevalence is reported to be between 94.9% and 96.4% in pregnant women [19, 24]. Especially in children, migration background is well characterized as a factor associated with a higher CMV seroprevalence [15]. It remains unclear if the overall declining trend in CMV seroprevalence possibly continued in the subpopulation of patients without a migration background, as we did not have information on the ethnicity of our patients.

In the most recent decade (2009–2018), women had a significantly higher CMV seroprevalence than men (59.80 versus 50.82%), a surplus of 8.98%. Other studies also observed a higher seroprevalence in females [11, 13, 16]. Even though this phenomenon has been observed before, it is not entirely understood why women have a higher CMV seroprevalence. While social factors like earlier and more extensive involvement in the care of potentially infectious toddlers and infant might be contributing, this would not explain the higher CMV seroprevalence in females of all age groups. A sharp increase in CMV seroprevalence occurs in females between the age groups 10 to 19 and 20 to 29 years of age, and in males between the age groups 20 to 29 and 30 to 39 years of age (Fig. 4). The delayed increase in men, as well as the higher overall prevalence in women after puberty, might be attributed to CMV, like other sexually transmitted infections, being sexually transmitted at a higher rate from infectious men to susceptible women than from infectious women to susceptible men [25].

In women of childbearing age presenting to the Department of Obstetrics and Gynecology, we observed a CMV seroprevalence of 65.95%, which is significantly higher than the overall rate of 59.80%. There was no significant change during the last 30 years (Fig. 4). Other studies in women of childbearing age in Germany reported lower rates in pregnant women, ranging from 34% in a small group of 34 pregnant women from all over Germany [11] to 46–52%, with data from a central German University Hospital [11, 18]. Stratified by age group and spanning over three decades, we could see the highest CMV seroprevalence in young women (76.22% in the age group 16 to 20 years of age) that was declining up to the group 31 to 35 years of age. This phenomenon has previously been observed in studies from 1991 (Friese et al.) and 2011 (Enders et al.) [16, 17], and might be due to women with lower socioeconomic status [26] as well as women with migration background from non-EU countries [27] tending to give birth to their first child earlier in life, both factors associated with higher CMV prevalence [16, 18, 28]. In the following age groups, CMV seroprevalence rises continuously (Fig. 4).

In patients with a known HIV infection, a further increase in the already high CMV seroprevalence could be observed, most strikingly in women, who had a CMV seroprevalence of 94.61%, an increase of 6.81% when compared with the former decade. In males, CMV seroprevalence also increased, but not quite as much, and was 92.36%. Especially in women with a known HIV infection, this trend might be associated with migration from Sub-Saharan Africa after 2013 [29], where CMV seropositivity in asymptomatic, HIV-positive patients approaches 100% [10]. It is noteworthy that the median value of all positive samples was higher in the group of patients that are known to be HIV positive, with a value of 2409 AU/mL, as compared with 1400 AU/mL in the HIV-negative group. It needs to be taken into account that this does not represent the mathematical mean, which could not be calculated due to a recorded upper limit in the AU/mL value, and it is unclear if this less than twofold difference represents a clinically relevant gap. In patients that could be identified as oncological, a CMV seroprevalence of 60.64% (*n* = 2091) was determined in the current decade. In pediatric patients, it was 51.60%, and 61.70% in adult patients. The excess from the overall seroprevalence of 56.48% in the HIV-negative cohort is statistically significant (*p* = 0.0002), but there are no numbers available in the literature to which we could compare this result of all combined oncological patients. The main goal of this study was the description of changes in CMV seroprevalence over time. In the case of oncological patients, however, we cannot compare this number to the previous decades, as the structures of our University Hospital as well as the data accompanying the samples has changed in the last 30 years, and no congruent groups could be defined. Patients could also not be divided into further subgroups of interest, such as solid organ or stem cell recipients, since such data was not easily attained. This is an area that should be further examined in future studies.

Other limitations of our study include our hospital cohort setting in an urban region of Germany, which is not representative of the general population. We also cannot be sure that the sent-in practices for CMV assays in our university hospital did not change during the examined period of 30 years, and influenced the comparability of the studies. During the last three decades, there was an increased awareness of the dangers of primary CMV infection during pregnancy, and therefore, more testing occurred in pregnant women, distorting the overall numbers by over-representing this group.

The strength of this study lies in the large number of patients (31,401 in the decade 2009–2018), and the good comparability of the test results, as the same diagnostic test had been applied over the last three decades.

Overall, the former significant decline of CMV seroprevalence between the decades 1988–1997 and 1998–2008 in this urban region of Germany slowed down to a non-significant decrease of 0.77% (1998–2008 vs. 2009–2018). This might be an indicator that the dynamic of the CMV seroprevalence has reached a plateau. It remains unclear whether the herein described changes in CMV immunity over time have a tangible impact on the incidence of CMV disease in groups at risk. In women of childbearing age, there was no significant change in CMV seropositivity in the last three decades, and therefore, no change in the proportion of women who are at risk of primary CMV infection during pregnancy.
